# P-1502. Clindamycin and *Group A Streptococcus*: Is It Time To Say Goodbye?

**DOI:** 10.1093/ofid/ofae631.1671

**Published:** 2025-01-29

**Authors:** Keeret Mann, Ritika Zijoo, Richard G Lugar

**Affiliations:** Indiana University School of Medicine/Ball Memorial Program, Muncie, Indiana; Indiana University Health, Muncie, Indiana; IU Health Ball Memorial Hospital, Fishers, Indiana

## Abstract

**Background:**

Group A streptococci (GAS) can cause invasive disease leading to Toxic Shock Syndrome (TSS), a condition linked with high mortality. Initial antibiotic therapy for GAS-TSS includes beta-lactams and clindamycin. When clindamycin resistance is noted, linezolid may be used instead until clinical stability is achieved. We reviewed GAS susceptibility at IUH Ball Memorial Hospital to assess appropriateness of initial clindamycin use as adjunctive therapy for GAS-TSS.

**Figure:**
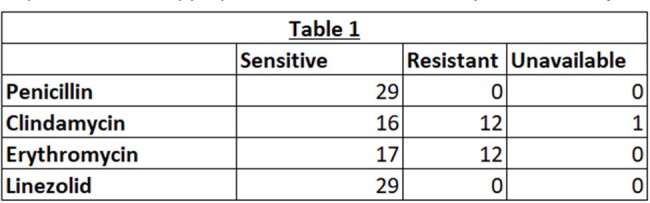

**Methods:**

GAS positive cultures recovered from 1/1/23 – 12/31/23 at IU Health Ball Memorial Hospital, Muncie, IN were reviewed.

**Figure:**
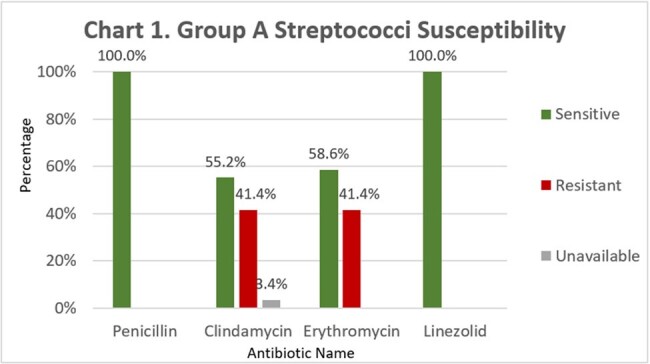

**Results:**

Out of 162 cultures positive for GAS, 29 (17.9%) had susceptibility results. All 29 GAS specimens were sensitive to penicillin and linezolid, but only 16/29 (55.2%) specimens were sensitive to clindamycin. Table 1. and Chart 1. show susceptibility results for penicillin, clindamycin, erythromycin, and linezolid. Table 2. shows number of specimens with inducible clindamycin resistance. Chart 2. shows various sources of culture specimens.

**Figure:**
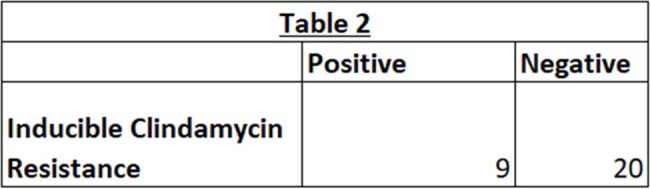

**Conclusion:**

GAS susceptibility testing was performed with invasive infections or when requested. Significant resistance was noted to clindamycin. Although our data is limited by a small sample size, it is in line with trends noted by US CDC’s Active Bacterial Core surveillance reports. This raises concern over initial use of clindamycin for TSS before susceptibilities are available. There are both advantages and disadvantages associated with use of clindamycin or linezolid. There is more data to support clindamycin use, however it is linked with increased risk of C. difficile infections. There is limited data for the use of linezolid for this indication, but it does maintain better susceptibility and negates the use of vancomycin at time of empiric therapy. Even though more data may be needed to definitively recommend linezolid over clindamycin or vice versa, clinical decisions will need to be made prior to susceptibility results. We recommend clinicians utilize their local antibiograms to guide decision making, while also accounting for patient factors. This also provides an opportunity for the development of clinical practice guidelines to better assist clinicians in the community.

**Figure:**
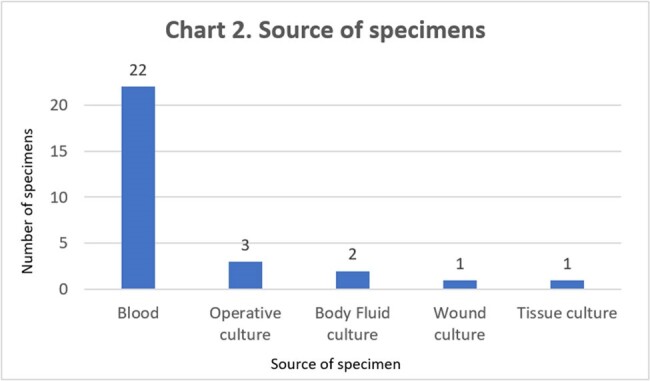

**Disclosures:**

**All Authors**: No reported disclosures

